# General population preferences for cancer care in health systems of China: A discrete choice experiment

**DOI:** 10.1002/cam4.5473

**Published:** 2022-12-08

**Authors:** Nan Zhang, Xuan Chang, Ruyue Liu, Caiyun Zheng, Xin Wang, Stephen Birch

**Affiliations:** ^1^ Center for Health Management and Policy Research, School of Public Health, Cheeloo College of Medicine, Shandong University Jinan China; ^2^ NHC Key Lab of Health Economics and Policy Research (Shandong University) Jinan China; ^3^ Shandong Cancer Hospital and Institute, Shandong First Medical University and Shandong Academy of Medical Sciences Jinan China; ^4^ Department of Publicity Shandong Provincial Hospital Affiliated to Shandong First Medical University Jinan China; ^5^ School of Public Health Weifang Medical University Weifang China; ^6^ School of Public Health Sun Yat‐Sen University Guangzhou China; ^7^ Centre for the Business and Economics of Health, University of Queensland Brisbane Australia

**Keywords:** cancer care, discrete choice experiment, integrated network, preference

## Abstract

**Background:**

The increasing incidence of cancer in China has posed considerable challenges for cancer care delivery systems. This study aimed to determine the general population's preferences for cancer care, to provide evidence for building a people‐centered integrated cancer care system.

**Methods:**

We conducted a discrete choice experiment that involved 1,200 participants in Shandong Province. Individuals were asked to choose between cancer care scenarios based on the type and level of hospitals, with various out‐of‐pocket costs, waiting time, and contact working in the hospitals. Individual preferences, willingness to pay, and uptake rate were estimated using a mixed‐logit model.

**Results:**

This study included 848 respondents (70.67%). Respondents preferred county hospitals with shorter hospitalization waiting times and contact working in hospitals. Compared to the reference levels, the three highest willingness to pay values were related to waiting time for hospitalization (¥97,857.69–¥145411.70–¥212,992.10/$14512.70–$21565.16–$31587.61), followed by the county‐level hospital (¥32,545.13/$4826.58). The preferences of the different groups of respondents were diverse. Based on a county‐level general hospital with contact in the hospital, 50% out‐of‐pocket costs and a waiting time of 15 days, the probability of seeking baseline care was 0.37. Reducing the waiting time from 15 to 7, 3, and 0 days, increases the probability of choosing a county‐level hospital from 0.37 to 0.58, 0.64, and 0.70, respectively.

**Conclusions:**

This study suggests that there is a substantial interest in attending county‐level hospitals and that reducing hospitalization waiting time is the most effective measure to increase the probability of seeking cancer care in county‐level hospitals.

## Graphical abstract

This study suggests that there is a substantial interest in attending county‐level hospitals and that reducing hospitalisation waiting time is the most effective measure to increase the probability of seeking cancer care in county‐level hospitals.

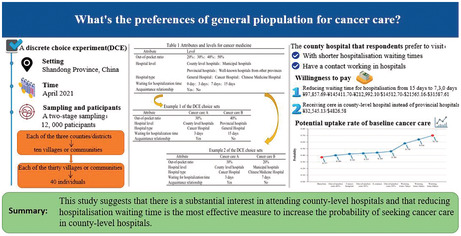

## 1 INTRODUCTION

Cancer was the leading or second leading cause of death in 112 countries in 2019, according to the World Health Organization.^1^ China has experienced rapid increase in cancer incidence and mortality in China over the past four decades. The percentage of deaths caused by cancer as a proportion of all deaths in the country increased from 10.1% in 1975 to 24.2% in 2015.^2^, ^3^ It was estimated that there would by 4.57 million new cancer cases in China in 2020, accounting for approximately 23.7% of the global cancer incidence.^4^ Moreover, the high cancer incidence has placed a high economic burdens on health systems and families. The direct medical cost of cancer in China was estimated to be ¥221.4 billion ($32.83 billion) in 2015, accounting for 5.4% of total health expenditures.^5^ In 2014, a hospital‐based multicenter cross‐sectional survey revealed that the 1‐year out‐of‐pocket expenditure of a newly diagnosed colorectal cancer patient accounted for 59.9% of annual household income and caused 75.0% of families to suffer an unmanageable financial burden.^6^

With increasing incidence rates and economic burdens, cancer has brought considerable challenges to the health systems of China. Since the 1980s, these systems have been characterized by a disease‐focused hospital‐centered approach with a weak primary care system. First, there were significant gaps in cancer prevention and control, relating to health protection policies, primary prevention, secondary prevention, and access to early detection and treatment. In 2014–2015, only 12.1% of the 16,394 respondents in a multicenter cross‐sectional survey conducted in 16 provinces had undergone cancer screening.^7^ Second, in the absence of a system organized around primary care gate‐keepers and access to hospital care by referral from primary care, many cancer patients sought care for cancer in higher‐level hospitals with lower health insurance reimbursements and limited health workforce, resulting in rapid increases in medical costs and low system efficiency. A 2017 community‐based cross‐sectional survey of 527 patients with any of the six cancers in 15 cities revealed that between 49% and 76% of patients sought care in tertiary hospitals whereas between 19% and 40% of them sought care in secondary hospitals.^8^ The average hospitalization cost per visit for cancer patients was ¥17,567 ($2605.26) with between 12 and 17 days in tertiary hospitals.^8^ The payment for cancer care from health insurance funds to health institutions within the county accounted for 0.94% of the global payment for cancer care. However, these institutions were responsible for screening, follow‐up, and rehabilitation.^9^

These challenges demonstrate the need for innovative solutions in Chinese health care systems, particularly the development of stronger systems for providing cancer care services through an integrated network. In the past 15 years, both national and local health commissions have introduced new measures. First, the Chinese government funded four cancer screening programs to increase access to early detection and treatment.^10^, ^11^ Second, the Chinese government established the National Cancer Registration and Follow‐up Programme in 2008 to measure the cancer burden in the population and provide evidence for policy makers to set priorities for the allocation of cancer control resources for the prevention, diagnosis, and treatment of cancer.^12^ Third, since 2012, several cancer prevention and control action/plans in China have been issued, including plans for 2012–15, 2015–17, 2017–25, and 2019–2022.^13^, ^14^ The plans emphasized building five‐level (county‐city‐province‐region‐nation) integrated cancer care networks, especially for constructing county‐level cancer hospitals.

People‐centered care is an approach that consciously adopts the perspectives of individuals, families, and communities, and views them as participants as well as beneficiaries of trusted health systems that are responsive to their needs and preferences.^15^ In building a five‐level integrated cancer care network, population preferences are crucial determinants of uptake and adherence to best practice care. Discrete choice experiments (DCEs) have been utilized to elicit and quantify population and patients' preferences for health plans, vaccination programs, and health providers' preferences for employment.^16^, ^17^, ^18^ In the case of cancer care, most studies have focused on patients' preferences for treatment plans, follow‐up plans, or hospice plans, in the settings of individual hospitals or clinics.^19^, ^20^ Some studies have explored population preferences for screening, such as the method of screening and providers who provide the screening, generating evidence for early cancer detection.^21^, ^22^ From the perspective of a multilevel health system with multitype hospitals and various health policies, there is a knowledge gap in jointly considering the effect of care characteristics and related policies on the general population's potential health‐seeking behavior.

Shandong province is located in eastern China. As reported in 2020, it has the second largest population (1,015.27 million) and the fourth largest percentage of residents aged 65 and older (13.99%) among all provinces of China.^23^ In response to the national cancer prevention and control plan, and the increasing demands of the local population, Shandong Province is establishing an integrated cancer care delivery system,^24^ especially the strengthening capacity of county hospitals. This study aimed to elicit the preferences of the general population for different cancer care configurations and to predict the probability of individuals seeking cancer care in county‐level hospitals, thereby providing evidence about the current practice in Shandong health systems.

## 2 MATERIALS AND METHODS

### 2.1 Study setting

The research was conducted in China's Shandong Province. Three counties were randomly sampled from the eastern, middle, and western areas of this province. None of the three counties contained county or district level cancer hospitals. Cancer screening, diagnosis, and treatment are provided by county or district level general hospitals, city‐level general hospitals and cancer hospitals, province‐level general hospitals and cancer hospitals. Previously, it was estimated that the average out‐of‐pocket cost for cancer patients during the first 3 years after diagnosis, including outpatient and inpatient costs at all levels and types of hospitals was ¥38,772 in Shandong province, accounting for 32%–35% of the total cost ¥120,000 ($17796.50).^25^

### 2.2 DCE development

#### 2.2.1 Selection of attributes levels

According to Janssen et al,^26^ there are six methods for instrument development in choice experiments. In this study, three of them were combined in a stepwise manner, namely literature review, expert consultation, and pre‐test interview. The literature review revealed that nine factors influence patients' choices in low‐ and middle‐income countries: self‐reported severity, previous medical history, having a contact working in the hospitals, distance between home and hospital, waiting time for hospitalization, type and level of the hospitals, being treated friendly, health outcomes, and medical expenses.^27^, ^28^, ^29^ In expert consultations with 12 oncologists from county/city/province‐level hospitals, self‐reported severity and health outcomes were excluded. Compared to patients with common diseases, it is difficult for patients with cancer to report their condition's severity. Similarly, it is challenging for doctors and patients to identify short‐term outcomes following the treatment of certain cancers. In expert consultations with six professors involved in the management and delivery of cancer care, distance to the hospitals was eliminated as an attribute because it is dependent on another attribute, the levels of hospitals. Administrative areas define the levels of hospitals and geographic location determines the division of administrative areas. Therefore, the level of hospitals can represent the distance to hospitals to some extent. During the pre‐test interviews with 10 residents, some indicated that they might value friendliness of providers and the proximity to the hospitals as relatively low. This is because cancers are recognized as severe diseases such that the individual prefers more prolonged survival than unfriendly treatment. Table 1 presents the levels of the five attributes in the final survey.

**TABLE 1:** Attributes and levels of cancer care

Attribute	Level	Definition
Out‐of‐pocket cost	¥24,000/$3559.30 (20% of total cost^a^)	The average out‐of‐pocket cost for cancer patients during the first 3 years after diagnosis, including outpatient and inpatient costs in all levels and types of hospitals
	¥36,000/$5338.95 (30% of total cost^a^)	
	¥48,000/$7118.60 (40% of total cost^a^)	
	¥60,000/$8898.25 (50% of total cost^a^)	
Hospital level	County hospital	Hospital classification according to China's current ‘Hospital Classification Management Measures’ and other regulations
	Municipal hospital	
	Provincial hospital	
	Famous hospitals from other provinces	
Hospital type	General hospital	Hospital classification according to the services provided by the hospital and the scope of received patients
	Cancer hospital	
	Chinese Medicine hospital	
Waiting time for hospitalization	0 days	The waiting time from the issuance of the inpatient bill to the actual admission to the hospital (i.e., waiting time for bed)
	3 days	
	7 days	
	15 days	
A contact working in the hospital	Yes	Someone you know working in the hospital who helps you or gives you information
	No	

^a^ The total cost is ¥120,000 ($17796.50).

#### 2.2.2 Experimental design and choice task development

We created a D‐efficient, labeled, factional factorial design using Ngene1.1.2, and generated 24 choice sets. Each choice set consisted of alternatives A and B for which the attribute levels varied systematically. Twenty‐four choice pairs were distributed randomly across the three versions of the questionnaires, to avoid overloading respondents' capacities to complete the survey.^30^ The three versions of the questionnaires were randomly allocated to the respondents.

#### 2.2.3 Validity

To examine the respondents' understanding and attention, the first choice set was duplicated and inserted as the fifth choice set in each questionnaire version. Furthermore, we assessed the response rates, attribute dominance, and self‐reported evaluations to determine the validity of the results. On a 5‐point Likert scale ranging from *strongly agree* to *strongly disagree*, participants were asked “Do you understand the above options in Part 2 of the questionnaire?”

### 2.3 Sampling and data collection

In stated‐preference methods, sample size calculations are based on rules of thumb, and the recommended minimum sample size are 300 or 500.^31^, ^32^, ^33^ We targeted a sample size of 1000 to be sufficient to guarantee precision in the estimation of all the model parameters. Invitations for participation in the survey were sent to 1,200 individuals in anticipation of a potentially large non‐response rate. Then, a two‐stage sampling procedure was conducted. First, 10 villages or communities were randomly sampled from the village/community list in each of the three counties/districts. Second, a simple random sampling of 40 individuals were selected from the population registry of 30 villages or communities. To assess preference differences across subgroups, this study included all individuals aged 40–70 (representing a population at high risk for cancer), willing to participate, able to provide written informed consent, and able to complete the questionnaire, including those with cancer history.

In addition to the nine choice sets, the questionnaire also included an explanation of the terms used and the sociodemographic information of respondents. In the pilot survey of 12 respondents aged 40~60, suggestions were made regarding the use of simple and respondent‐friendly language. Responses indicated that the participants understood the choice sets and could make trade‐offs among the two alternatives. In April 2021, the final survey was conducted in 30 villages/communities while COVID‐19 was under control and patient travel was not restricted. Participation in the survey was voluntary, with a consent form, and the participants were not compensated. To improve the questionnaire response rate and quality, doctors in villages and general practitioners in community health stations assisted the research team in locating and visiting the sampled 1,200 individuals. Trained investigators assisted the respondents in completing the questionnaire. Additionally, two researchers (NZ and XW) randomly interviewed eight respondents (2 respondents aged 40–49, 2 aged 50–59, 2 aged 60–69, and 2 over 70) in each county/district to determine the reasons underlying their choices. This study complied with the Declaration of Helsinki and the ethical approval was obtained from the Institutional Ethics Review Board of Shandong Cancer Hospital and Institute (Reference No.SDTHEC201909001).

### 2.4 Data analysis

STATA 15.1 was used for statistical analyses. Using MAXQDA 11, a thematic analysis of interviews with 24 respondents was conducted.

DCE is based on the random utility theory assumption, which assumes that the utility associated with a good or service comprises the utilities of its characteristics.^34^ The utility acquired by individual n from alternative j can be expressed as follows:

$$U_{\mathrm{nj}}=V_{\mathrm{nj}}+\varepsilon_{\mathrm{nj}}\;j=1,\ldots \ldots ,J$$

- where $U_{\mathrm{nj}}$ consists of the systematic component $V_{\mathrm{nj}}$ and a random component $\varepsilon_{\mathrm{nj}}$. The systematic component $V_{\mathrm{nj}}$ is a function of the observed healthcare attribute $x_{\mathrm{nj}}$ and the observed individual characteristics n. The random component $\varepsilon_{\mathrm{nj}}$ is related to unobserved attributes or preference variation.

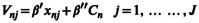

- where $C_{n}$ represents the individual's characteristics n, $\beta^{\prime }$, and $\beta^{\prime \prime }$ are vectors of the coefficients to be estimated.

#### 2.4.1 Regression and willingness to pay

The relative importance of the attributes and levels in the DCE was estimated using regression analysis. Model selection between conditional and mixed logit regression was made by comparing the Akaike and Bayesian information criterion.^35^, ^36^ The estimated coefficients in the regression model provide information regarding the direction and significance of the effects of changing the levels of an attribute. However, they could not provide a valuation required for comparing alternatives. Therefore, we calculated the willingness to pay (WTP) additional out‐of‐pocket cost to receive a higher level of a specific cancer healthcare attribute. Estimating the WTP for an attribute or level x can be done as

-
$$\mathrm{WTP}\left(x\right)=\frac{\partial U/\partial x}{\partial U/\partial \mathrm{opt}}$$
- where opt denotes out‐of‐pocket costs. In addition, using all survey respondents, we conducted subgroup analyses based on different age groups, gender, family cancer history, and annual family income groups to elicit specific preferences of the subgroups.

#### 2.4.2 Change of uptake rate

Policymakers in hospitals and health commissions are interested in how the probability of a respondent choosing a particular type of healthcare varies as the attribute levels change. To provide evidence to inform the decision to build a county‐level cancer hospital in Shandong Province, we conducted a simulation study to explore the potential uptake of attending the county‐level hospital as service attributes change. The random utility theory assumes that an individual chooses the alternative with the highest level of utility. When individual n is asked to choose between alternatives i and j, the probability of choosing alternative i is given by:

-
$$\begin{array}{cc} P_{\mathrm{ni}}\; & =\text{Prob}\left(U_{\mathrm{ni}}>U_{\mathrm{nj}}\right)\\ & =\text{Prob}\left(V_{\mathrm{ni}}+\varepsilon_{\mathrm{ni}}>V_{\mathrm{nj}}+\varepsilon_{\mathrm{nj}}\right)\\ & =\text{Prob}\left(V_{\mathrm{ni}}-V_{\mathrm{nj}}>\varepsilon_{\mathrm{nj}}-\varepsilon_{\mathrm{ni}}\right)\forall i\neq j \end{array}$$

When the random component $\varepsilon_{\mathrm{nj}}$ is assumed to be an independent and identically distributed extreme value, the probability that individual n chooses alternative i can be estimated as follows:

$$P_{\mathrm{ni}}=\frac{e^{v_{\mathrm{ni}}}}{\sum_{j}e^{V_{\mathrm{nj}}}}=\frac{e^{\left(\beta_{x_{\mathrm{ni}}}^{\prime }+\beta \prime \prime C_{n}\right)}}{\sum_{j}e^{\left(\beta_{x_{\mathrm{nj}}}^{\prime }+\beta \prime \prime C_{n}\right)}}$$

- where X is a vector of the attribute coefficients. If the policies (levels) related to alternative i are altered, the probability of receiving healthcare using the previously preferred alternative k would change as follows:
-
$$\text{Impact of policy change}=P_{\mathrm{ni}}-P_{\mathrm{nk}}\;i\neq j$$

## 3 RESULTS

### 3.1 Respondents

Of the 1,200 invited participants, 964 provided informed consent of which 80.33% completed the survey. A total of 116 respondents failed the consistency test, because they made different choices in the repeated choice set. Sensitivity analyses, which excluded respondents who failed the consistency test, did not alter the results of the analyses. Among the 116 respondents who failed the consistency test, 17.24% lacked confidence to comprehend their choices. Meanwhile, only 3.06% of the 848 respondents who passed the consistency test lacked confidence. There was also a statistical significance in the rate of passing the consistency test among the three counties. Of the 848 respondents who passed the consistency test, four always chose “alternative A” and one always chose “alternative B” among all choice tasks. Hence, we excluded 116 respondents and remained with 848 respondents for further analysis.

Table 2 presents the respondents' characteristics. The mean age was 57.44 years. Most respondents were female (2/3), married (95.64%), and had no family history of cancer (80.19%). Nearly half of the participants reported having undergone cancer screening. Nearly two‐thirds of the respondents had an annual family income of ¥30,000 ($4449.12) or less, which is less than the average out‐of‐pocket health care cost (¥38,772 or $5750.05) of cancer patients in Shandong Province during the first 2–4 years after diagnosis.

**TABLE 2:** Characteristics of respondents

Characteristics	Respondents *n* = 848 (who passed the consistency test)		Non‐Respondents *n* = 116 (who failed the consistency test)		*χ* ^2^	*p*
	*n*	%	*n*	%		
Age(year), mean ± SD	57.44 ± 7.11		57.31 ± 7.96			
Gender						
Male	291	34.32	39	33.62	0.0219	0.882
Female	557	65.68	77	66.38		
Age(year)						
40–49	119	14.03	21	18.10	2.9740	0.226
50–59	389	45.87	44	37.93		
60–69	340	40.10	51	43.97		
Marital status						
With a partner	811	95.64	111	95.69	0.0007	0.979
Without a partner	37	4.36	5	4.31		
Annual family income(¥/$)						
<¥10,000/$1483.04	187	22.05	25	21.55	1.7831	0.410
¥10,000–29,999/$1483.04–4448.98	358	42.22	56	48.28		
≥¥30,000/$4449.12	303	35.73	35	30.17		
Location						
County A	313	36.91	22	18.97	19.5538	0.000
County B	263	31.01	57	49.14		
County C	272	32.08	37	31.89		
Family history of cancer						
Yes	168	19.81	20	17.24	0.4293	0.512
No	680	80.19	96	82.76		
Screening for cancer						
Ever	462	54.48	71	61.21	1.8673	0.172
Never	386	45.52	45	38.79		

### 3.2 Preferences for cancer care attributes and willingness to pay of all respondents

We selected the mixed logit model for regression instead of the conditional logit model, based on the goodness of fit. Table 3 displays the regression results and monetary valuation for each attribute and level, as perceived by all respondents. Except for Chinese Medicine Hospital for the attribute “Hospital type”, coefficients and levels of all other attributes are statistically significant. Respondents preferred county hospitals, especially county cancer hospitals, with shorter hospitalization waiting times and contact working in the hospital. The coefficient relating to renowned hospitals in other provinces is negative, implying that respondents prefer to receive cancer care locally within the province. The estimated willingness to pay for changes in attribute levels was highest for changes in waiting time for hospital admission (¥212,992.10/$31587.61, ¥145411.70/$21565.16, ¥97,857.69/$14512.70). They were at least three times the estimated WTP amounts or any other change in attribute level change. In addition to hospitalization waiting time, respondents were willing to pay ¥32,545.13 ($4826.58) for county‐level hospitals, ¥26840.48 ($3980.55) for a contact working in the hospital, ¥21,176.11($3140.50) for treatment at a cancer hospital. Respondents were willing to receive cancer care in famous hospitals from other provinces if they received a ¥107,749.80 ($15979.74) subsidy, which is more than twice the average out‐of‐pocket cost (¥38,772/$5750.05) of cancer patients in Shandong Province during the first 3 years after diagnosis.

**TABLE 3:** Regression results and willingness to pay for cancer care (all respondents)

Attributes and levels	Coefficient means	Robust S.E.	Willingness to pay (¥)	95%CI (¥)
Constant	0.009	0.052	/	/
Hospital level: Provincial hospitals (ref)				
County level hospitals	0.419**	0.105	32545.13	13968.42–51121.84
Municipal hospitals	0.240**	0.085	18597.34	4399.67–32795.00
Famous hospitals in other provinces	−1.386**	0.124	−107749.80	−149093.50‐‐66406.06
Hospital type: General hospital (ref)				
Cancer Hospital	0.272**	0.068	21176.11	7801.36–34550.86
Chinese Medicine Hospital	0.115	0.063	8920.30	−1329.14‐19169.74
Waiting time for hospitalization: 15 days (ref)				
0 days	2.739**	0.145	212922.10	140243.50–285600.80
3 days	1.870**	0.108	145411.70	95498.37–195,325
7 days	1.259**	0.088	97857.69	64116.02–131599.30
A contact: No (ref)				
Yes	0.345**	0.057	26840.48	14738.64–38942.33
Out‐of‐pocket	0.0000129**	2.22 e‐06	/	/

Abbreviations: ref, reference which reflects a reference level for each attribute; /, not applicable.

** *p* < 0.01.

### 3.3 Subgroup analysis of preferences for cancer care attributes and willingness to pay

Tables 4 and 5 analyses preferences for cancer care attributes and willingness to pay in the different groups. The most significant difference in priorities among different age groups is that younger respondents (40–49 years old) placed greater importance on receiving cancer care in provincial hospitals and paid no attention to out‐of‐pocket costs. Compared with no subsidy in provincial hospitals, only if a double subsidy of the out‐of‐pocket costs makes them consider cancer care in county hospitals. There are different preferences for cancer care between male and female participants. The estimated coefficients of all attributes and their levels were statistically significant for females. However, the estimated male coefficients were not statistically significant for hospital level (inside the province), hospital type, or contact.

**TABLE 4:** Regression results and WTP for cancer care (subgroup analysis: age & gender)

Attributes	Age (year)						Gender			
	40–49 (*n* = 119)		50–59 (*n* = 389)		60–70 (*n* = 340)		Male (*n* = 291)		Female (*n* = 557)	
	Coefficient	WTP (¥)	Coefficient	WTP (¥)	Coefficient	WTP (¥)	Coefficient	WTP (¥)	Coefficient	WTP (¥)
Constant	−0.091	/	−0.030	/	0.073	/	−0.166	/	0.080	/
Hospital level: Provincial hospitals (ref)										
County level hospitals	−0.707*	−507792.40	0.499**	45897.81	0.885**	44108.39	0.072	6283.94	0.642**	48400.60
Municipal hospitals	−0.192	−137891.40	0.215	19824.21	0.559**	27884.00	0.170	14781.49	0.316**	23822.35
Famous hospitals in other provinces	−1.170**	−839760.20	−1.455**	−132973.70	−1.529**	−76236.85	−1.991**	−173538.80	−1.151**	−86853.91
Hospital type: General hospital (ref)										
Cancer Hospital	0.295	212028.10	0.418**	38476.90	0.149	7439.80	0.124	10851.36	0.362**	27287.81
Chinese Medicine Hospital	0.134	96542.26	0.152	13994.12	0.047	2431.08	−0.091	−7972.09	0.202**	15217.45
Waiting time for hospitalization: 15 days (ref)										
0 days	2.622**	1882482.00	2.851**	262298.00	2.970**	148050.40	2.788**	243016.70	2.803**	211437.30
3 days	1.624**	1166296.00	2.019**	185763.40	2.048**	102108.20	1.933**	168472.90	1.917**	144602.20
7 days	1.262**	906462.10	1.304**	120001.40	1.340**	66788.01	1.511**	131698.00	1.172**	88445.61
A contact: No (ref)										
Yes	0.403**	289793.30	0.282**	25942.74	0.460**	22915.24	0.187	16260.94	0.437**	32948.15
Out‐of‐pocket ratio	−1.39 e‐06	/	−0.0000109**	/	−0.0000201**	/	−0.0000115**	/	−0.0000133**	/

Abbreviations: ref, reference which reflects a reference level for each attribute; /, not applicable.

* *p* < 0.05

** *p* < 0.01.

**TABLE 5:** Regression results and WTP for cancer care (subgroup analysis: location & annual family income)

Attributes	Location						Annual family income					
	County A (*n* = 322)		County B (*n* = 263)		County C (*n* = 272)		<¥10,000/$1483.04 (*n* = 187)		¥10,000–29,999/$1483.04–4448.98 (*n* = 358)		≥¥30,000/$4449.12 (*n* = 303)	
	Coefficient	WTP (¥)	Coefficient	WTP (¥)	Coefficient	WTP (¥)	Coefficient	WTP (¥)	Coefficient	WTP (¥)	Coefficient	WTP (¥)
Constant	−0.053	/	0.072	/	0.001	/	−0.035	/	0.076	/	−0.043	/
Hospital level: Provincial hospitals (ref)												
County level hospitals	0.471**	36893.07	−0.175	−13192.99	0.974**	62593.14	0.815**	44906.22	0.799**	65121.68	−0.327	−36227.15
Municipal hospitals	0.221	17278.16	0.178	13409.02	0.417*	26766.45	0.315*	17363.55	0.556**	45263.77	−0.104	−11536.89
Famous hospitals in other provinces	−1.272**	−99592.50	−1.344**	−101547.90	−1.763**	−113282.30	−1.084**	−5.9708.73	−1.452**	−118300.10	−1.607**	−178226.10
Hospital type: General hospital (ref)												
Cancer hospital	0.112	8746.76	0.475**	35854.03	0.248	15913.08	0.210*	11570.59	0.172**	14032.56	0.487**	53997.40
Chinese medicine hospital	−0.108	−8422.78	0.313*	23657.74	0.194	12468.18	0.120	6604.87	0.072	5899.632	0.133	14733.25
Waiting time for hospitalization: 15 days (ref)												
0 days	1.777**	139144.10	4.224**	319118.80	3.189**	204882.40	2.545**	140213.10	2.631**	214304.80	3.141**	348358.50
3 days	1.227**	96103.67	2.834**	214051.80	2.221**	142708.80	1.918**	105671.30	1.690**	137631.60	2.103**	233255.50
7 days	0.822**	64384.75	1.947**	147075.20	1.411**	90653.34	1.252**	68967.60	1.178**	95944.98	1.427**	158249.20
A contact: No (ref)												
Yes	0.216**	16877.32	0.366**	27675.91	0.451**	29007.60	0.356**	19635.20	0.298**	24283.05	0.404**	44854.90
Out‐of‐pocket ratio	−0.0000128**	/	−0.0000132**	/	−0.0000156**	/	−0.0000181**	/	−0.0000123**	/	‐902 e‐06	/

Abbreviations: ref, reference which reflects a reference level for each attribute; /, not applicable.

* *p* < 0.05

** *p* < 0.01.

Respondents with a family history of care put less weight on the hospitals level within the province and contact working in the hospitals than those with no family history. However, they were willing to pay much more to reduce the waiting time. The preferences of respondents from the three counties regarding waiting time for hospitalization and out‐of‐pocket costs were comparable. Regarding levels of hospitals within the province, respondents from county C have significant preferences among all three levels, respondents from county A have no preference between provincial‐ and municipal‐level hospitals, while respondents from county B have no preference among hospitals within the province. Moreover, respondents from county B prefer a type of hospital that is different from that of respondents from the other two counties. Respondents from the two groups with an annual family income of less than ¥30,000 ($4449.12) have similar preferences for cancer care. While, respondents with over ¥30,000 ($4449.12) annual family income had no preference among county‐, municipal‐, and provincial‐level hospitals.

### 3.4 Potential uptake rate

**FIGURE 1:**
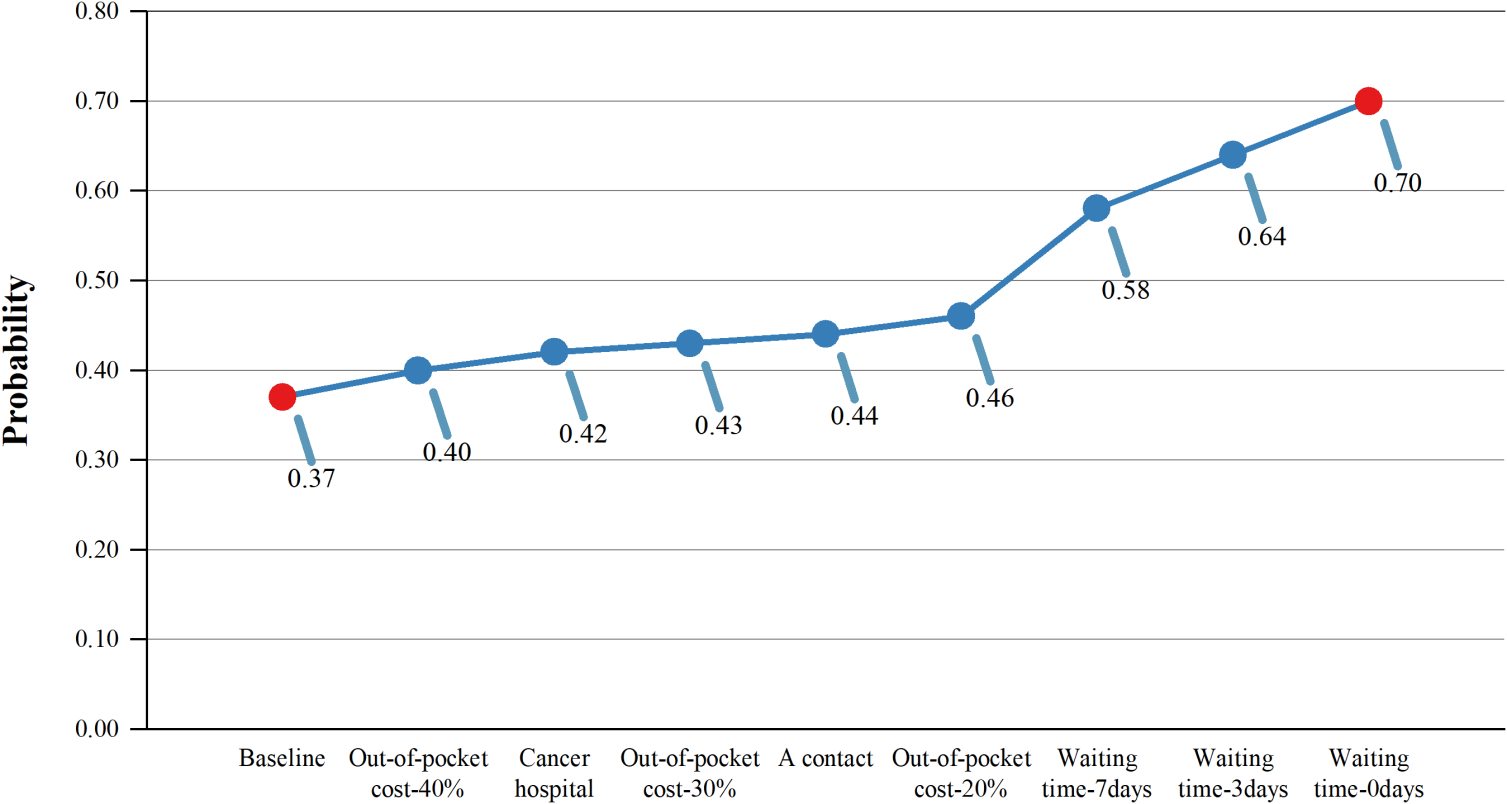
Probabilities of seeking cancer care in county hospitals

To provide evidence for the construction of county‐level cancer hospitals, we calculated the potential uptake rate. As shown in Figure 1, the probability of seeking baseline cancer care was 0.37, which was a county‐level general hospital with contact working in the hospital, 50% out‐of‐pocket costs, and a waiting time of 15 days. If a cancer hospital replaces a county‐level general hospital, the probability of seeking care in the county increases to 0.42. Reducing the out‐of‐pocket rate from 50% to 40%, 30%, and 20% increases the probability of choosing county‐level hospitals from 0.37 to 0.40, 0.43, and 0.46%, respectively. Shortening the waiting time from 15 to 7 days, 3 days, and 0 days increases the probability of choosing county‐level hospitals from 0.37 to 0.58, 0.64, and 0.70, respectively.

## 4 DISCUSSION

This study measures the relative importance of the general population assigned to each attribute of cancer care in terms of their willingness to pay and calculates how choice probabilities of county‐level hospitals vary with changes in attribute levels. Considering the general population's perceptions is essential for building a people‐centered integrated cancer care system.

### 4.1 Main findings and comparison with other literature

All five attributes significantly affected the respondents' choices. Regarding the levels of each attribute, respondents prefer county hospitals, especially county cancer hospitals, with shorter waiting times for hospitalization and having contact working in the hospital. Respondents preferred hospitals in their provinces to famous hospitals outside the province. Inside the province, they have no significant preference for Chinese Medicine hospitals, and prefer county hospitals the most. Waiting time for hospitalization is the most important characteristic for those in both the whole and the subgroup analysis, with the largest estimated out‐of‐pocket cost that the respondents are willing to pay. Waiting times were also significant attribute for colorectal cancer screening and breast cancer follow‐up services.^37^ Nevertheless, it did not play an important role in this study. The difference in these findings is mainly due to the different stages of cancer and the type of care. In this study, cancer care was identified as integrated care after the individual was diagnosed for the first time, including treatment, follow‐up services, and other related medical care. The more important role of waiting time in China than in other countries lies in unplanned patient flow. Most Chinese hospitals did not schedule the appointments, and the hospitals' unplanned patient flow was clogged.^38^ Moreover, without appointments, patients always have a high expectation of a short waiting time.

Subgroup analysis found that younger respondents had no preference among different out‐of‐pocket costs, and they preferred province‐level hospitals with more out‐of‐pocket costs to county and municipal hospitals, which is consistent with the preference of respondents in the higher annual family income group. According to demographic information and interviews with respondents, this is partly because that younger respondents aged 40–49 reported higher annual family income (¥42,278.00/$6270.00) than the older respondents (¥21,654.00/$3211.38). The effect of income on preference for out‐of‐pocket costs or the price of healthcare has also been demonstrated in other studies in China^39^, ^40^ and other countries.^41^, ^42^ Female respondents have significant preferences for hospital level, hospital type, and having contact working in the hospitals. This could provide evidence for allocating health care resources for cancer treatments targeting female or male subjects. Subgroups based on location showed preference heterogeneity in terms of the hospital type and level. Respondents in county B had a significant preference for cancer hospitals regardless of hospital level, and respondents from the other two counties preferred county‐level hospitals, regardless of hospital type. A study also showed different preferences for different therapies in young women in eight OECD countries.^43^ The different preferences of respondents in the three counties are a combination of hospital cultures and capacity, care provider attitudes and behavior and respondents' knowledge of cancer care.

Reducing waiting times for hospitalization may be an effective measure with the greatest WTP to increase the percentage of cancer care delivery by county‐level hospitals. A decrease in the out‐of‐pocket cost ratio could, to some extent, increase the probability of patients seeking cancer care in county‐level hospitals. Having contacts working in hospitals also influences respondents' choices and may provide a more effective method to influence individual care‐seeking behavior than establishing a cancer hospital. The importance of a contact in health‐seeking behavior has been revealed in previous studies.^44^ In a survey of the general population in Shandong province, 51.72% of the respondents indicated a preference for hospitals with a contact. If they had serious illness, the others said they would try to find a contact through referrals from friends or relatives.^45^ Fei proposed that the basic form of rural interpersonal relationships is the acquaintance society, which is determined by traditional rural production and lifestyle.^46^ The acquaintance society in Shandong Province, which has developed agriculture and vast rural areas, is deeply rooted than in other provinces in China. Therefore, having contact working in the hospital considerably affects respondents' preferences in Shandong Province.

### 4.2 Policy implication

This study's principal findings provide evidence for building a people‐centered integrated cancer care delivery system. First, in a multilevel cancer care network, it is necessary to strengthen the first level of contact (county). Depending on the preferences of respondents, the existing oncology departments of county‐level general hospitals in counties A and C, as well as the newly built county‐level cancer hospital in county B could be first‐level organizations for cancer care delivery. Second, allocating more resources for cancer care, targeting females, and county‐level hospitals are more likely to increase the participation of patients. Females prefer to receive cancer care at county‐level hospitals. Organizing cancer care centers for cervical, breast, and other female cancers at county‐level hospitals would help address the preferences of female patients. Third, measures to shorten hospitalization waiting time would affect patient participation or uptake. Collaboration among different levels, such as referring patients to county or district‐level hospitals for follow‐up services or rehabilitation, could increase patient throughput and shorten the average waiting time for hospitalization. Fourth, basing the doctor–patient relationship on a contact relationship using a gatekeeping system would assist in addressing patients' preferences. In 2011, Ke recognized the importance of having contact in healthcare delivery and proposed a relationship change through general physicians (GP).^47^ The contact relationship between GPs in community health centers and oncology doctors in county hospitals would help patients build trust and relationship with the oncology doctors. Finally, establishing a cross‐level medical consortium and promoting homogenization of cancer care in it would avoid seeking care in provincial hospitals or famous hospitals in other provinces and reduce medical expenditures.^48^ As stated in the respondents' interviews, their preferences for the hospital level are based on the assumption that the quality of care in higher‐level hospitals is better than that in lower‐level hospitals. Therefore, promoting homogeneous care for cancer and renaming multilevel institutions uniformly within the consortium would eliminate the general population's ‘preconception’ at the hospital levels.

### 4.3 Strengths and limitations

This is the first stated preference study to explore general population preferences for integrated cancer care in multilevel care delivery systems. The study design, DCE, quantifies the relative importance of the various attributes and levels. The sampling method, comprising multicentered clustered sampling of villages and community‐based randomized sampling of people ensured the collection of a representative sample. However, there are three limitations to this study. First, similar to other DCE studies, we investigated the stated preferences of the general population without a cancer diagnosis. Further research should compare the results with the revealed preferences based on patients' actual behavior. These distinctions would also be helpful for evidence‐informed policy making. Second, the DCE did not include some other important attributes. In order to make DCE manageable, we only included five attributes based on the literature review results, expert consultation, and pre‐test interview. Further research could adopt other important attributes, such as the quality of care, and compare the results. Third, caution should be exercised when this study's results and policy implications are extended to other health systems in China or other countries. These results are a combination of the health system background, health resources, people's demands, and other factors of the three counties. However, the DCE design and the adopted attributes, especially having contact working in the hospital, are applicable to other health care settings.

## 5 CONCLUSION

People's needs and preferences are central features of people‐centered integrated care. This study elicited the preferences of the general population for cancer care, calculated willingness to pay for the preferred attributes and levels of care, and predicted the likelihood that cancer patients will seek treatment in county‐level hospitals. Based on the findings, strengthening first‐level (county‐level) care in the multilevel cancer care network, arranging and allocating more resources related to female cancers in county‐level hospitals, taking measures to shorten waiting times for hospitalization, transforming doctor‐patient relationships into contact relationships by gate‐keeping systems, and establishing cross‐level medical consortia, and promoting homogenization of cancer care would be crucial strategies for increasing the uptake of cancer care. This study contributes to the development of policies for constructing people‐centered hierarchical multilevel cancer care delivery networks in Shandong and provides a methodology for investigating the general population's preferences for cancer care in other health care systems.

## AUTHOR CONTRIBUTIONS

**Nan Zhang:** Conceptualization (lead); formal analysis (equal); funding acquisition (equal); writing – original draft (equal). **Xuan Chang:** Conceptualization (supporting); data curation (equal); formal analysis (equal). **Ruyue Liu:** Data curation (equal); formal analysis (equal); investigation (lead). **Caiyun Zheng:** Data curation (equal); formal analysis (equal); investigation (supporting). **Xin Wang:** Conceptualization (equal); methodology (equal); supervision (equal); writing – review and editing (lead). **Stephen Birch:** Conceptualization (supporting); methodology (equal); writing – review and editing (equal).

## FUNDING INFORMATION

This study was supported by the National Natural Science Foundation of China (71904109).

## CONFLICT OF INTEREST

There is no conflict of interest to disclose.

## ETHICAL APPROVAL

This study complied with the Declaration of Helsinki, and the ethical approval was obtained from the Institutional Ethics Review Board of Shandong Cancer Hospital and Institute (Reference No.SDTHEC201909001).

## Data Availability

The data supporting this study's findings are available from the corresponding author upon reasonable request.
